# Phylotastic: Improving Access to Tree-of-Life Knowledge With Flexible, on-the-Fly Delivery of Trees

**DOI:** 10.1177/1176934319899384

**Published:** 2020-04-06

**Authors:** Van D Nguyen, Thanh H Nguyen, Abu Saleh Md Tayeen, H Dail Laughinghouse, Luna L Sánchez-Reyes, Jodie Wiggins, Enrico Pontelli, Dmitry Mozzherin, Brian O’Meara, Arlin Stoltzfus

**Affiliations:** 1 Department of Computer Science, New Mexico State University, Las Cruces, NM, USA; 2 Institute for Bioscience and Biotechnology Research, Rockville, MD, USA; 3 Fort Lauderdale Research and Education Center, University of Florida/IFAS, Davie, FL, USA; 4 Department of Ecology and Evolutionary Biology, The University of Tennessee, Knoxville, Knoxville, TN, USA; 5 Illinois Natural History Survey, Species File Group, University of Illinois at Urbana–Champaign, Champaign, IL, USA; 6 Office of Data and Informatics, Material Measurement Laboratory, NIST, Gaithersburg, MD, USA

**Keywords:** Phylogeny, tree of life, web services

## Abstract

A comprehensive phylogeny of species, i.e., a tree of life, has potential uses in a variety of contexts, including research, education, and public policy. Yet, accessing the tree of life typically requires special knowledge, complex software, or long periods of training. The Phylotastic project aims make it as easy to get a phylogeny of species as it is to get driving directions from mapping software. In prior work, we presented a design for an open system to validate and manage taxon names, find phylogeny resources, extract subtrees matching a user’s taxon list, scale trees to time, and integrate related resources such as species images. Here, we report the implementation of a set of tools that together represent a robust, accessible system for on-the-fly delivery of phylogenetic knowledge. This set of tools includes a web portal to execute several customizable workflows to obtain species phylogenies (scaled by geologic time and decorated with thumbnail images); more than 30 underlying web services (accessible via a common registry); and code toolkits in R and Python (allowing others to develop custom applications using Phylotastic services). The Phylotastic system, accessible via http://www.phylotastic.org, provides a unique resource to access the current state of phylogenetic knowledge, useful for a variety of cases in which a tree extracted quickly from online resources (as distinct from a tree custom-made from character data) is sufficient, as it is for many casual uses of trees identified here.

## Introduction

A phylogeny broadly covering the diversity of known species would provide “a comparative and predictive framework for all fundamental and applied biology.”^1^ Such a tree of life is not just a tool for researchers: like a detailed map of world geography, or a periodic table of the chemical elements, a tree of life is a fundamental guide to the living world, something to be consulted by policymakers, taught by educators at all levels, and explored by curious members of the public. Therefore, it is important for knowledge of the tree of life to be discoverable and accessible.

Yet, phylogenetics is traditionally a technical discipline with inaccessible products. Phylogenies are difficult to generate from raw data following best practices, which are constantly updated to reflect new methodologies and expanding sources of data. Phylogeny specialists typically do not employ practices that make their phylogenies discoverable, accessible, and re-useable.^2-4^

This situation is beginning to change, and the potential has emerged recently to make expert phylogenetic knowledge accessible for a broad array of uses. First, a supertree constructed from available taxonomies and published phylogenies is periodically updated by the OpenTree project.^5,6^ The current “synthetic tree” has more than 2 million species and is constructed from 987 source trees. Second, the practical value of this tree of life is greatly enhanced by the availability of systematic data from other resources, including images and basic taxon information (size, habitat, etc) from the Encyclopedia of Life (EOL),^7^ taxonomic name mappings,^8,9^ and occurrence records from the Global Biodiversity Information Facility (GBIF)^10^ or iNaturalist. Third, there has been a common movement among all of the above (and other) resource providers to support programmatic access to content via web services. A web service interface provides access to data or operations over the World Wide Web, using standard machine-to-machine communication protocols. The simplest web service queries can be typed manually into a web browser, but web services are designed to be interoperable, so that they can be invoked automatically by other software and chained together to create workflows.

The combination of these developments makes possible the kind of “Phylotastic” system envisioned previously.^11^ The Phylotastic design concept leverages an open system of web services to support various workflows to discover, modify, and add value to trees. The aim is to make it as easy to get a tree online as it is to get travel directions from a mobile app or transit system website.

Here, we describe the first full implementation of this design. To identify the most common scenarios for getting a quick tree (use cases), possibly combined with other data, we consulted a diverse set of potential users, emphasizing nonspecialists (thus complementing the analysis in Stoltzfus et al^11^). Based on the resulting list of desirable features, we designed and implemented more than 30 web services, a web service registry, libraries in R and Python, and a web portal. Together, these capabilities make the knowledge and insights provided by phylogenetic trees available to the myriad of users, from researchers to middle school students, who do not possess the technical expertise to develop their own trees. In particular, the Phylotastic web portal is an interactive web application that illustrates the capabilities of the system, supporting a variety of workflows for obtaining trees beginning with a list of taxa, which may be constructed by various means, e.g., scraping names from electronic documents or websites. The resulting trees, which have thumbnail images and links to catalog entries, may be saved as images or data. The underlying web services that provide this functionality may also be accessed using custom software, with the aid of the R and Python libraries.

## Description

### Analysis and design

The concept for a Phylotastic system^11^ grew from the research interests of scientists with expertise in phylogenetics and informatics. To ensure broader value to the community, we developed a prototype Phylotastic web portal and used it to obtain feedback (via correspondence as well as in-person interviews) from a broader range of potential users, including scientists and educators at various levels who are not experts in informatics or phylogenetics. Based on this information, we prioritized the development of tools and workflows to support the following use cases.

1. Generate a tree from a specified list of taxa. Provide a tree for a user-supplied list of species or higher taxa.
2. Generate a tree of N species sampled from a named taxon. Given a named taxon and a number N, provide a tree with N species chosen in some way, e.g., at random, by popularity, or by maximal diversity (taxonomic or phylogenetic).
3. Generate a phylo-guide from an electronic resource. Create a tree with images and links to species information from a resource that includes taxonomic names, such as a scientific paper, a website about ants, or a document listing the species found in a park or zoo.
4. Contextualize phylogenetic relationships. Place a given list of species in a larger tree that shows phylogenetic relationships in a broader context. Or, given a small set of taxa, generate a tree using representative species that illustrate the relationship, possibly including species from other (unspecified) taxa for context.
5. Integrate data or metadata with phylogeny. Given the set of species implicated by any method described above, return a tree and an associated data table integrating information or resources of interest, including images, links to information (e.g., EOL or Wikipedia), or data on features such as toxicity, pathogenicity, availability of fossil data, medicinal value, conservation status, size, biogeography, or habitat.

Currently, the system we implemented covers each use case partially or wholly (see Examples and Discussion). Some types of data requested by potential users (e.g., availability of fossil data), and some types of operations, are not yet implemented in available algorithms (e.g., choosing a set of species based on both popularity and diversity).

### Implementations

The types of operations identified previously for the implementation of a Phylotastic system^11^ include (1) taxonomic name resolution, i.e., rectifying possible misspellings in scientific names by matching them to authoritative taxonomic data bases; (2) tree retrieval, i.e., finding available trees with coverage of user-identified taxa and extracting subtrees; (3) tree scaling, i.e., assigning branch lengths to subtrees; (4) tree comparison, i.e., comparing subtrees; (5) taxon information and images, i.e., getting and adding data or metadata from species or higher taxa in the subtree; (6) rendering a tree graphically. The expanded set of use cases identified above implicates a slightly larger set of operations: (7) scraping names, i.e., extracting taxonomic names embedded in text and media; (8) taxon sampling, i.e., choosing species from a taxonomic group by some criteria; (9) converting common names to scientific names; and (10) list management, i.e., creating, reading, publishing, updating, and removing a list of names associated with a managed user account.

*Web services*: The above operations were translated to actual tools (services) that can be accessed and manipulated by users and that are implemented and executed via web services written in Python. In general, Phylotastic web services are designed to operate synchronously. This means that workflows are carried out in real time. One exception is the set of services to manage persistent lists, so that a list created by a client in a session may be accessed in a later session, or by a different client, enhancing the potential for reusability. Currently, we have made available more than 30 web services described in Table 1. Some of these services are thin wrappers around external services, while others were developed for this project. Documentation for Phylotastic web services can be accessed via the web portal; source code is available from the Phylotastic project on GitHub.

**Table 1.** Main Phylotastic services description.

Web service	Description
Common Names to Scientific Names	Get scientific name of a species from common name
NCBI_common_name	following the NCBI database
EBI_common_name	following EBI services
ITIS_common_name	following ITIS services
TROPICOS_common_name	following TROPICOS services
EOL_common_name	following EOL services
Scientific Name Extraction	Scrape scientific names from URL, text or file
GNRD_wrapper_URL	using Global Names Recognition and Discovery
GNRD_wrapper_text	using Global Names Recognition and Discovery
GNRD_wrapper_file	using Global Names Recognition and Discovery
TaxonFinder_wrapper_URL	using Global Names Recognition and Discovery
TaxonFinder_wrapper_text	using Taxon Finder
Taxonomic Name Resolution	Match scientific names and resolve mismatches
OToL_TNRS_wrapper	using the Open Tree of Life taxonomy
GNR_TNRS_wrapper	using the Global Names Resolver tool
iPlant_TNRS_wrapper	using iPlant collaborative services
Taxon Sampling	Get scientific names within a taxon, including
Taxon_all_species	all scientific names
Taxon_country_species	those found in a given country (iNaturalist),
Taxon_genome_species	those having a genome sequence (in NCBI),
Taxon_popular_species	the most popular species using OneZoom
Taxon Information and Images	Get various information of a species such as
Image_url_species	image URLs and license information using EOL
Info_url_species	information URLs from EOL
ECOS_Conservation	conservation status from ECO services
Tree Retrieval	Get phylogenetic trees from a list of taxa
OToL_wrapper_Tree;	from Open Tree of Life synthetic tree
OToL_supported_studies	and all supporting studies
Phylomatic_wrapper_Tree	from Phylomatic
Treebase_Tree	from TreeBase
Supersmart_wrapper_Tree	using supersmart
Tree Scaling	Scale branch lengths of a tree to geologic time
Datelife_scale_tree	using the DateLife service
OToL_scale_tree	using OToLs unofficial scaling service
Tree Comparison	Compare phylogenetic trees
Compare_trees	symmetrically
List Management	Manage taxon lists
Add_new_list; Remove_list	add or remove list of names
Replace_species_list	replace some names with supplied names
Update_metadata_list	update the metadata for a list
Get_list;	retrieve a list

Abbreviations: EBI, European Bioinformatics Institute; EOL, Encyclopedia of Life; ITIS, Integrated Taxonomic Information System; NCBI, National Center for Biotechnology Information.

*Code toolkits*: We developed R^12^ and Python packages to allow users to access the Phylotastic system with their own software and computers, using functions and methods written in the native language. Both toolkits provide access to nearly all the categories of services described in Table 1. The rphylotastic package wraps Phylotastic web services using the R packages jsonlite^13^ and httr,^14^ designed for working with URLs and HTTP calls, with roxygen2^12^ for documentation and testthat^15^ for automated tests.

**Figure 1.**
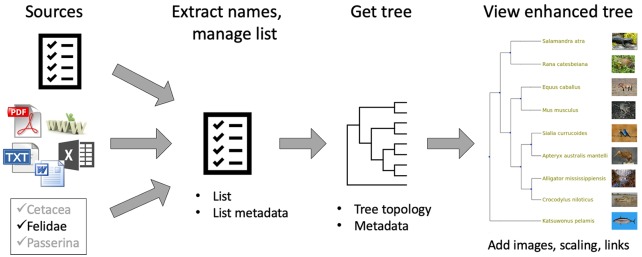
A simplified view of workflows supported by the Phylotastic portal (see text for explanation). A variety of methods are used to generate a list of taxa, which can be used to query for a tree topology. The topology can be scaled and decorated with images and links. The portal provides a built-in viewer for phylogenies, which may be downloaded as an image file or a Newick tree file.

The phylotastic_py package has a main module, phylotastic_services composed of submodules implementing the different Phylotastic services. The package documentation was generated with Sphinx.^16^ To test the functional correctness of submodules, a set of unit tests were implemented using Python Unit Testing Framework and deployed in Travis-CI (https://travis-ci.org/). *Web portal*: The Phylotastic portal (http://portal.phylotastic.org) provides a graphical interface to create or select a list of taxa, manage the list, and retrieve a tree, which is displayed using an embedded viewer. Figure 1 provides a simplified illustration of workflows supported by the portal. The user may populate a list of taxa by choosing a public list already on the portal, by uploading a list, by extracting taxonomic names from an electronic resource (PDF, xls, doc, txt, and html) that is uploaded or identified by a URL, or by using methods to select species from a named taxon (at random, by popularity, by genome entries in the National Center for Biotechnology Information (NCBI), from location records at iNaturalist). Although the resulting list of verified taxon names may be downloaded, a more typical step is to request a tree. The initial tree, from OpenTree, is a topology only, but it may be decorated with images and scaled using DateLife.^17^

The portal manages sessions and accounts so that users can maintain persistent lists and trees. Lists can be downloaded in a simple format with 1 name per line. Trees can be downloaded as Newick or as a graphical rendering in the png or Scalable Vector Graphics (SVG) format. These features allow the portal to support a wide range of use cases, as explained below (Examples).

The web portal is written in Ruby using Rails, a model-view-controller architecture for rapid design and development of robust web applications. The portal takes advantage of PostgreSQL for database management; Paperclip for managing file attachments; TwitterBootstrap, JQuery, and FontAwesome for front-end development; Devise for authentication management; Wicked PDF for PDF generation; Capybara and Minitest for automated testing; and Docker and Kubernetes for containerization and deployment. The test suite covers model tests, controller tests, and interactive tests (simulated in Poltergeist, which mimics user interactions).

*Web services registry*: The portal and library software described above depend on concrete workflows instantiated by specific known services. However, if web services are described abstractly (e.g., in terms of input types, options, and output types) in a web service registry using a machine-readable language, then it is possible for an intelligent system to query the registry, discover useful services, and construct a workflow on the fly. Indeed, the Phylotastic project was designed with the aim of supporting this kind of automatic workflow construction, and all the web services above are described abstractly in the registry. The use and importance of the Phylotastic web services registry in automated workflow composition, and in fault-tolerant execution (e.g., re-computing a workflow to avoid an unresponsive service), has been thoroughly described elsewhere.^18,19^

## Examples

Each of the use cases described above is supported, partly or fully, by the Phylotastic portal. The first 3 use cases (user-supplied list, sample from taxon, generate phylo-guide) are straightforward applications of the web portal’s workflow of designating a list, managing the list, and retrieving a tree for visualization. Because lists can be downloaded in a simple format with 1 name per line, it is straightforward to combine or edit lists manually. This makes it possible to embed a list of focal species in a sample from a higher taxon (use case 4), or to construct the example used in Figure 3 (aquatic mammals), explained in more detail below.

The portal integrates a limited set of useful data and metadata (use case 5), specifically EOL links and thumbnail images. One way to integrate other data by combining the portal and external graphical tools is to (1) use the portal to scrape names from a data table (in text or Excel format) and then obtain and download (as a Newick file) a phylogeny for the implicated taxa, then (2) combine the original data table and the downloaded tree using a web tool designed for this purpose, such as EvolView^20^ or Interactive Tree Of Life (IToL).^21^

For users who are able to write code in Python or R, much more flexibility is possible using the toolkits described above. Each of the use cases above is supported, at least partly, by the toolkits. An example of contextualizing a list of species within a larger taxon (use case 4) is given in detail below.

### Birds from Yellowstone

In this use case, a visitor to Yellowstone National Park composes a list of birds seen in the park and then uses Phylotastic tools to (1) translate the common names to scientific names, (2) get all scientific names of Yellowstone birds from the US National Park Service website (https://www.nps.gov/), and (3) get a dated phylogeny of all species and plot them with observed species highlighted, as shown in Figure 2. The scaled tree is constructed from curated trees via DateLife,^17^ without any need for the user to conduct phylogenetic inference or calibration. The format of the Park Service list does not matter because names are scraped using intelligent algorithms. This example can be executed as follows using the DateLife and R-Phylotastic packages:

library("rphylotastic", "datelife")

# 1. start with user’s list of birds from Yellowstone:

birds_I_saw <- taxa_common_to_scientific(c("Osprey",

"House sparrow", "Mallard duck", "American Robin",

"Song Sparrow", "Mourning Dove", "House Wren"))

# 2. combine this with NPS’s list of all Yellowstone birds:

yellowstone_birds <- url_get_scientific_names(

URL="https://www.nps.gov/yell/learn/nature/upload/

BirdChecklist2014.pdf")

# 3. get the tree with dates

yellowstone_bird_tree <- datelife::datelife_search(

taxa_get_otol_tree(yellowstone_birds), summary_format

= "phylo_median")

# 4. plot as Fig. 2 (plot code: see rphylotastic vignette)

**Figure 2.**
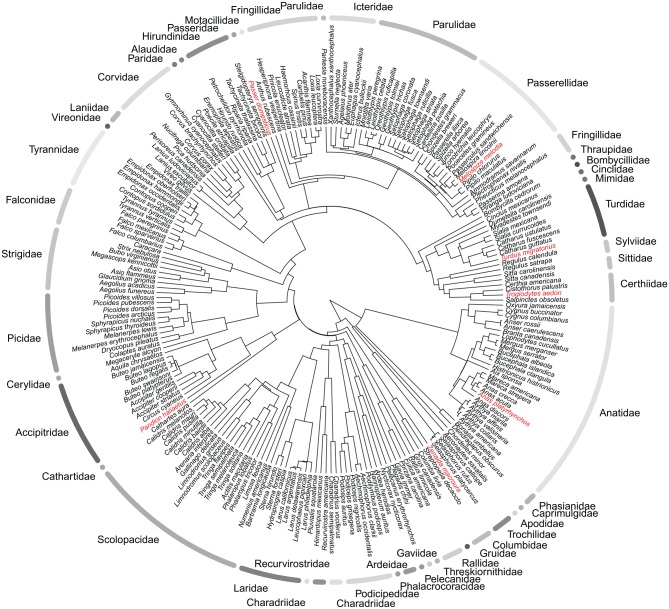
Dated phylogenetic tree of birds from Yellowstone National Park obtained as described in the text. Species from the user’s observation list are shown in red. Bird families are delimited by gray arcs. This figure was generated with functions from rphylotastic, DateLife, and ape^22^ R packages. The complete code for this vignette is in the rphylotastic package.

### Aquatic mammals

**Figure 3.**
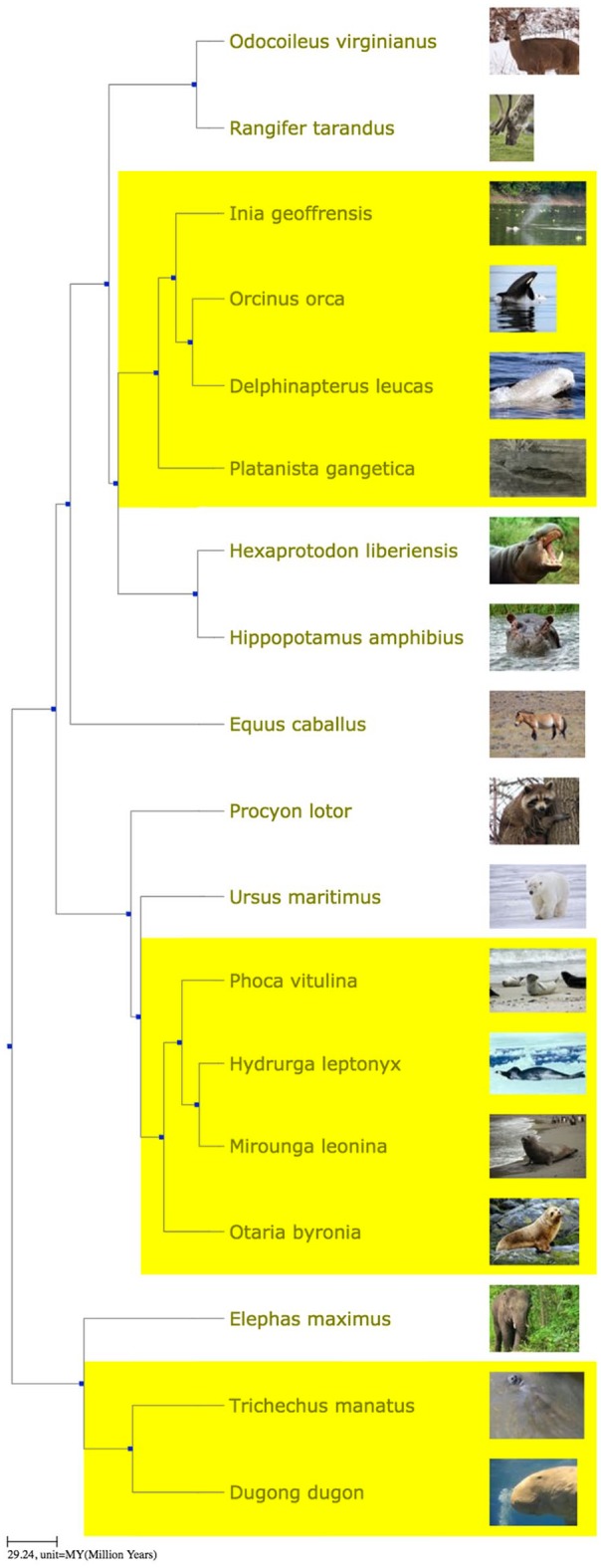
Three separate groups of aquatic mammals (Cetacea, Pinnipedia, and Sirenia). This figure was generated using the Phylotastic portal, as described in the text.

The portal screencast (www.youtube.com/watch?v=8Q5m0ldaGIg) illustrates the ability to upload a PDF, extract names, and generate a tree with images and links. In this case, the source is Tsagkogeorga et al,^23^ a scientific publication about the origin of Cetacea (whales and dolphins). The portal extracts 39 taxon names from the PDF, including 26 species names. Retrieving a tree yields a tree with 26 tips including whales, dolphins, and various mammalian outgroups. The screencast explains how to expand this into a lesson about repeated evolution, by including representatives of 2 other aquatic groups, with relatives of each. Specifically, the list of species scraped from the PDF is downloaded, some names are removed to simplify the presentation, and then, a small list of names is added for the Pinnipedia (the seals and sea lions) with their carnivore relatives, and the Sirenia (the dugongs and manatees) with an elephant as close relative. To obtain Figure 3, a time-scaled tree from this list is decorated with thumbnail images, and the 3 groups are highlighted using the clade-highlighting feature of the portal’s tree viewer.

## Discussion

### Comparison with other resources

Phylotastic resources can be considered broadly as a way of making tree-of-life knowledge accessible conveniently to nonspecialists, and as a way of supporting automated workflows to get phylogenetic knowledge and mash it up with other information. The system is designed for automation, integration, and convenience. As such, it is unique. Ordinary approaches of phylogenetic inference are out of reach for most users, even online systems that, from the perspective of experts, provide convenient interfaces (e.g., CIPRES, Phylogeny.fr). The Tree of Life Web Project (ToLWeb),^24^ OneZoom,^25^ IToL,^21^ and the OpenTree web portal all allow interactive browsing of a tree of life, and OpenTree and TreeBase both provide some web services. Several taxonomically limited phylogeny resources are available for vertebrates (see https://vertlife.org/data/), birds,^26^ primates,^27^ and seed plants^28^, each with its own particular way to get the tree (request by email, website, etc). However, the Phylotastic project, via either the portal or the suite of web services (accessed directly or via toolboxes), provides a unique functionality. For instance, combining automated tree retrieval with a diverse set of powerful methods to generate lists of interest to users is a combination that makes the Phylotastic project unique, and this combination only partly captures the capacity of the portal or the suite of web services.
